# Stability of grain zinc concentrations across lowland rice environments favors zinc biofortification breeding

**DOI:** 10.3389/fpls.2024.1293831

**Published:** 2024-02-13

**Authors:** Mbolatantely Rakotondramanana, Matthias Wissuwa, Landiarimisa Ramanankaja, Tantely Razafimbelo, James Stangoulis, Cécile Grenier

**Affiliations:** ^1^ Rice Research Department, The National Center for Applied Research on Rural Development (FOFIFA), Antananarivo, Madagascar; ^2^ Crop, Livestock and Environment Division, Japan International Research Center for Agricultural Sciences (JIRCAS), Tsukuba, Japan; ^3^ PhenoRob Cluster and Institute of Crop Science and Resource Conservation (INRES), University of Bonn, Bonn, Germany; ^4^ University of Antananarivo, Antananarivo, Madagascar; ^5^ College of Science and Engineering, Flinders University, Bedford Park, SA, Australia; ^6^ Centre de coopération internationale en recherche agronomique pour le développement (CIRAD), Amélioration génétique et adaptation des plantes méditerranéennes et tropicales (UMR AGAP Institut), Montpellier, France; ^7^ UMR AGAP Institut, Univ Montpellier, CIRAD, Institut national de recherche pour l'agriculture, l'alimentation et l'environnement (INRAE), Institut Agro, Montpellier, France; ^8^ Alliance Bioversity-Centro Internacional de Agricultura Tropical (CIAT), Cali, Colombia

**Keywords:** *Oryza sativa*, baseline zinc concentration, genotype by environment interactions, grain yield, Zn biofortification breeding targets, stability

## Abstract

**Introduction:**

One-third of the human population consumes insufficient zinc (Zn) to sustain a healthy life. Zn deficiency can be relieved by increasing the Zn concentration ([Zn]) in staple food crops through biofortification breeding. Rice is a poor source of Zn, and in countries predominantly relying on rice without sufficient dietary diversification, such as Madagascar, Zn biofortification is a priority.

**Methods:**

Multi-environmental trials were performed in Madagascar over two years, 2019 and 2020, to screen a total of 28 genotypes including local and imported germplasm. The trials were conducted in the highlands of Ankazomiriotra, Anjiro, and Behenji and in Morovoay, a location representative of the coastal ecosystem. Contributions of genotype (G), environment (E), and G by E interactions (GEIs) were investigated.

**Result:**

The grain [Zn] of local Malagasy rice varieties was similar to the internationally established grain [Zn] baseline of 18–20 μg/g for brown rice. While several imported breeding lines reached 50% of our breeding target set at +12 μg/g, only few met farmers’ appreciation criteria. Levels of grain [Zn] were stable across E. The G effects accounted for a main fraction of the variation, 76% to 83% of the variation for year 1 and year 2 trials, respectively, while GEI effects were comparatively small, contributing 23% to 9%. This contrasted with dominant E and GEI effects for grain yield. Our results indicate that local varieties tested contained insufficient Zn to alleviate Zn malnutrition, and developing new Zn-biofortified varieties should therefore be a priority. GGE analysis did not distinguish mega-environments for grain [Zn], whereas at least three mega-environments existed for grain yield, differentiated by the presence of limiting environmental conditions and responsiveness to improved soil fertility.

**Discussion:**

Our main conclusion reveals that grain [Zn] seems to be under strong genetic control in the agro-climatic conditions of Madagascar. We could identify several interesting genotypes as potential donors for the breeding program, among those BF156, with a relatively stable grain [Zn] (AMMI stability value (ASV) = 0.89) reaching our target (>26 μg/g). While selection for grain yield, general adaptation, and farmers’ appreciation would have to rely on multi-environment testing, selection for grain [Zn] could be centralized in earlier generations.

## 1 Introduction

Zinc (Zn) is an essential micronutrient for the growth and development of all living organisms including human beings (Broadley et al., 2007). Nevertheless, a significant portion—roughly one-third—of the global population does not ingest adequate amounts of zinc and therefore suffers from Zn deficiency (Brown et al., 2004). Zn deficiency particularly affects children and women, with related health problems such as stunting, loss of appetite, impaired immune function, diarrhea, eye and skin lesions, weight loss, delayed healing of wounds, and mental lethargy (Brown et al., 2004; Galetti, 2018). Zn deficiency and other micronutrient and vitamin deficiencies have been termed “hidden hunger” (Kennedy et al., 2003), and their alleviation is considered one of the top 10 priorities of humankind as defined in the Sustainable Development Goals (Goal 2.2: End all forms of Malnutrition).

Preventive supplementation of Zn and Zn fortification of processed foods have been used to reduce Zn deficiency-related problems but have had limited impact because of the recurring costs of providing a weekly or daily dose and because of the difficulty of delivering them in rural regions (Berti et al., 2014). Increasing the Zn content in the main staple food consumed daily by the targeted population, a concept termed Zn biofortification (Bouis, 2002; Brown et al., 2004; Bouis and Welch, 2010) would thus be a promising and cost-effective alternative. This approach is building on existing consumption patterns rather than attempting to change them. Typically, Zn biofortification is achieved through breeding for higher Zn concentrations in edible parts, but the application of foliar Zn fertilizers to the crop to achieve the same goal has been considered (Bouis and Welch, 2010). Developing crop varieties with enhanced Zn concentrations will not only reduce the number of severely malnourished people who require treatment by complementary interventions but also maintain an improved nutritional status in the longer term. Successful examples of released Zn-biofortified crops include maize, rice, wheat, and sorghum, but for rice, these cases are limited to Asia and Latin America (Andersson et al., 2017; Sanjeeva Rao et al., 2020; Virk et al., 2021), whereas no concerted effort has targeted the development of high-Zn rice varieties adapted to Africa to date.

Rice is not a good source of dietary Zn (Frossard et al., 2000) and may only provide one-fifth of daily Zn requirements (Sharma et al., 2013). This would be of little concern if the daily diet was augmented by the consumption of better Zn sources such as animal products milk, cheese, red meat, or seafood (Frossard et al., 2000). However, dietary diversification is frequently not practiced in resource-poor families that have insufficient funds to diversify their daily diet (Shahzad et al., 2014). Thus, countries with high rates of rice consumption and a high prevalence of poverty, like Sierra Leone, Guinea, and Madagascar, are ranked as the top 3 target countries for Zn biofortification in Africa (Herrington et al., 2019). Rice is the staple food in these countries, and in the case of Madagascar, it is eaten three times a day, providing 50%–80% of the daily caloric intake (WFP, 2016). Zn deficiency is a particular concern among children in the central highlands of Madagascar where a high prevalence of stunting (59.9%), wasting (6.0%), and underweight (40.1%) have been attributed to insufficient Zn intake (Shiratori and Nishide, 2018).

For a Zn biofortification breeding program to be successful, it is necessary to establish the baseline level of grain Zn concentrations ([Zn]) in varieties currently grown in farmers’ fields. To date, such data are not available for most African countries including Madagascar. However, based on the more extensive work performed in Asia, it is assumed that polished, white non-biofortified rice typically contains 16 μg/g Zn (Virk et al., 2021). To provide 40% of the daily requirement, it was recommended to target grain [Zn] of 28 μg/g in Zn-biofortified rice varieties (Bouis and Welch, 2010) (https://www.harvestplus.org/crop/zinc-rice). The long-term breeding goal has thus been set at +12 μg/g Zn above the baseline to alleviate Zn malnutrition in populations relying predominantly on rice in their daily diet. The distribution of Zn in the rice grain is relatively uniform, and removing the more nutrient-dense aleurone layer in polishing brown rice is only expected to reduce grain [Zn] between 15% and 20% (Sanjeeva Rao et al., 2020; Taleon et al., 2020). Thus, the breeding target of 28 μg/g Zn in white rice would correspond to approximately 34 μg/g Zn in brown rice.

When enhancing the grain Zn concentration of rice during the crop improvement process, it is essential to carefully consider other important traits. Among the numerous characteristics to be integrated into a breeding program, both grain yield and grain [Zn] are genetically complex traits (Norton et al., 2014; Babu et al., 2020; Rakotondramanana et al., 2022). Furthermore, it has been shown that agronomic management and environmental factors influence Zn uptake, translocation, and loading into grains and that seasonal effects possibly linked to water supply and soil redox state further affect [Zn] in grains (Goloran et al., 2019; Inabangan-Asilo et al., 2019). For grain yield, extensive genotype × environment interactions (GEI) are common in rice (Huang et al., 2021; Nguyen et al., 2023), and this necessitates multi-environment trials (METs) during variety development. Since GEI is only important when it causes significant changes in genotypic rankings in different environments, it is important for the efficacy of a breeding program to obtain estimates of the strength of GEI effects relative to genotype (G) and environment (E) effects. Through studies providing estimates for the relative strengths of G, E, and GEI effects, it will be possible to select genotypes that are stable across environments (Annicchiarico, 1997; Yan et al., 2000) or to identify genotypes specifically adapted to certain environments (Blanche et al., 2009).

As for any other trait, conventional breeding for Zn biofortification relies on the availability of genetic diversity within the compatible gene pool of the species. Screening of large germplasm collections has identified sources of high grain [Zn] in rice among varieties, landraces, or wild relatives of rice (Gregorio et al., 2000; Rakotondramanana et al., 2022; Senguttuvel P et al., 2023). Crossing these potential high-Zn donors with highly productive modern cultivars, followed by simultaneous selection for high micronutrient content and grain yield, will lead to the successful development of Zn-dense rice and the release of biofortified varieties for commercial cultivation (Garg et al., 2018; Senguttuvel P et al., 2023).

The effect of the environment on grain [Zn] is well established with factors such as soil and climate influencing average grain [Zn] at different sites (Wissuwa et al., 2008; Goloran et al., 2019). It is currently not known to what extent GEI plays an additional role and would have to be considered during the variety development process. Estimates of GEI for grain [Zn] obtained from studies conducted in Asia vary from as low as 1.9% (Suman et al., 2021) to 27.4% (Babu et al., 2020). Comparable data from Africa where rice is typically grown with few fertilizer inputs and where grain yields are lower compared to those from Asia (Saito et al., 2019) are not available. Madagascar is one of the countries severely struggling with micronutrient deficiency or hidden hunger (United Nations Children’s Fund (UNICEF), 2021). Furthermore, Malagasy farmers cultivate rice in very diverse environments, from hot and humid coastal plains to more temperate highland regions. With the goal of developing a lowland rice biofortification breeding program, a series of trials were conducted across a wide range of environments to assess the relative importance of G, E, and GEI effects on Zn concentrations in grains.

Given the scarcity of data available on Zn levels in rice for Madagascar, the objectives of this study were i) to determine baseline grain [Zn] in rice for Madagascar and to formulate breeding targets accordingly; ii) to select breeding lines combining high [Zn] with high grain yield in farmers’ fields in diverse rice-growing environments; iii) to assess the strength of G, E, and GEI effects on grain [Zn] and grain yield; and iv) to identify potential donors for the high-grain-Zn trait. To this end, multi-environmental trials (METs) were conducted at several sites in the central highland and coastal rice-growing regions of Madagascar using a set of Zn-biofortified breeding lines imported from the Centro internacional de agricultura tropical (CIAT, now “Alliance Bioversity-CIAT” in Colombia) in comparison to local and international check varieties.

## 2 Materials and methods

### 2.1 Evaluation sites and years

All rice genotypes (*Oryza sativa* L.) included in this study were evaluated in farmers’ fields at three locations in the Malagasy highlands at elevations between 980 m and 1,418 m, namely, Anjiro, Ankazomiriotra, and Behenjy, and one site located in the northwestern coastal region of Madagascar, Marovoay (**Supplementary Figure 1**). This study was carried out over a 2-year period in 2019 and 2020, with experiments in the highlands being conducted during the rainy season between November and May, whereas experiments at the coastal sites were conducted during the dry season from June to October (**Table 1**). All experiments were conducted under lowland conditions: a 3–8-cm layer of standing water was maintained in bunded fields throughout the crop cycle, through surface irrigation with water drawn from small local creeks and canals. Weeding was performed manually 3 and 8 weeks after transplanting, whereas pest control was not necessary. Soil samples were taken from each field prior to land preparation and sent to a central laboratory for analysis of pH, available P (Olsen), total N, and soil organic carbon (SOC).

**Table 1:** Environments used for the evaluation of grain yield and grain Zn concentrations during the year 1 (2019) and year 2 (2020) trials.

Year	Environments		Season	Latitude, Longitude	Altitude (masl)	Temp. (°C)	Rainfall (mm)	Sowing, harvesting (yyyymmdd)	pH (water)	Olsen P (mg/kg)	SOC (g/kg)	Total N (g/kg)
2019	Ankazomiriotra	ANK1	Main	19°66′45″S, 46°55′70″E	1016	21.3	1,198	20181107, 20190401	5.19	4.65	14.94	1.42
	Anjiro	ANJ1	Main	18°89′99″S, 47°97′46″E	980	23.4	1,059	20181108, 20190330	4.99	1.41	42.84	3.12
	Marovoay	MAR1a	Off	16°18′05″S, 46°67′80″E	10	26.3	51	20190514, 20190822	5.24	3.42	21.64	2.04
	Marovoay	MAR1b	Off	16°17′80″S, 46°68′58″E	10	26.3	51	20190514, 20190822	4.72	2.93	25.39	2.17
2020	Ankazomiriotra	ANK2	Main	19°67′93″S, 46°57′02″E	1016	21.7	1,082	20191030, 20200325	5.53	3.70	9.39	0.94
	Anjiro	ANJ2a	Main	18°90′56″S, 47°96′84″E	980	23.9	1,326	20191029, 20200408	5.17	4.08	22.45	1.87
	Anjiro	ANJ2b	Main	18°90′73″S, 47°97′30″E	980	23.9	1,326	20191030, 20200408	5.07	1.67	35.89	2.73
	Behenjy	BEN2a	Main	19°20′74″S, 47°48′20″E	1418	19.2	664	20191017, 20200312	5.26	1.66	16.56	1.22
	Behenjy	BEN2b	Main	19°24′59″S, 47°47′89″E	1418	19.2	664	20191016, 20200312	5.01	2.22	19.56	1.37
	Marovoay	MAR2a	Off	16°18′05″S, 46°67′80″E	10	25.4	53	20200628, 20201010	4.95	2.52	18.29	1.68
	Marovoay	MAR2b	Off	16°17′13″S, 46°66′87″E	10	25.4	53	20200628, 20201010	5.25	2.15	16.54	1.45

The main season corresponds to the rainy season in the highlands (November–May), whereas the off-season in the coastal region from June to October corresponds to the dry season (trials are conducted with irrigation). Average temperatures (Temp.) and cumulative rainfall are given for the period of rice cultivation.

### 2.2 Experimental materials

First-year trials were conducted with a common germplasm set of 24 lines. Of these, 22 were introduced to Madagascar from the CIAT rice breeding program. These were 20 Zn-biofortified elite breeding lines (BF-Lines) and two checks widely evaluated in all CIAT-HarvestPlus METs: the IR64 mega-variety, included as BF091, and the high-Zn check IR68144, evaluated in rice biofortification trials in Asia and Latin America. The remaining two lines were national check X265 and a second IR64 variant of IRRI origin (**Table 2**). National check X265 is the most commonly grown variety in the highland region of Madagascar, but at coastal sites, no dominant variety exists, and five additional local checks were included to obtain a more representative picture of grain Zn concentrations present in the coastal region.

**Table 2:** Germplasm tested in the year 1 (Y1) and year 2 (Y2) trials.

ID	Group	Designation	Year
BF001	BF-Line	CT19298-(100)-1-2-3-1-4MP	Y1
BF021*	BF-Line	IR31917-45-3-2-1-2SR-1-M	Y1/Y2*
BF035	BF-Line	CT23073-9-8-2	Y1
BF008	BF-Line	CT22061-1P-1SR-2P-3SR	Y1
BF011	BF-Line	CT22062-5P-3SR-1P-2SR	Y1
BF012	BF-Line	CT22062-5P-3SR-1P-3SR	Y1
BF014	BF-Line	CT22062-5P-3SR-2P-2SR	Y1
BF015	BF-Line	CT22062-5P-3SR-2P-3SR	Y1
BF045	BF-Line	CT22117-9P-1SR-2P-3SR	Y1
BF050	BF-Line	CT22118-1P-6SR-5P-1SR	Y1
BF051	BF-Line	CT22118-5P-1SR-1P-2SR	Y1
BF054	BF-Line	CT22129-2P-8SR-2P-1SR	Y1
BF055	BF-Line	CT22129-2P-8SR-2P-3SR	Y1/Y2
BF060	BF-Line	CT22135-9P-5SR-1P-3SR	Y1
BF109	BF-Line	CT23135-F4-61-M	Y1/Y2
BF110	BF-Line	CT23119-F2-7-1-3SR-3P	Y1/Y2
BF111	BF-Line	CT23110-F2-9-4-3SR-2P	Y1
BF153	BF-Line	CT22154-9P-1SR-1P-3SR	Y1
BF156	BF-Line	CT22128-5P-5SR-1P-3SR	Y1/Y2
BF105	BF-Line	CT23140-F4-38-M	Y1
BF091	BF-Line	IR64 from CIAT	Y1
IR68114	High-Zn check	IR68144-2B-2-2-3-1-166	Y1
X265	National check	X265	Y1/Y2
IR64	Mega-variety check	IR64	Y1/Y2
10114	IRIS_313-10114	FACAGRO_64	Y2
9368	IRIS_313-9368	CHANDARHAT	Y2
HP02	Local check coast	Tsipala_A	Y1
HP03	Local check coast	Sebota_281	Y1
HP04	Local check coast	Mahadigny	Y1
HP05	Local check coast	Varimanitra	Y1
HP06	Local check coast	Tsiresindrano	Y1
HP07	Local check highlands	Vary Botry	Y2
HP08	Local check highlands	Vary Mena	Y2
HP09	Local check highlands	Tsemaka	Y2
HP10	Local check highlands	Duralex	Y2
HP11	Local check highlands	X243	Y2

*Only included at coastal sites in year 2 trials.

National check variety X265 and mega-variety IR64 were grown at all sites, whereas local check varieties were specific to each location.

Agronomic trials in year 2 were conducted with a common set of eight lines. These were the four best-performing biofortified CIAT BF-Lines from year 1, national check X265, international check IR64 from IRRI, and two high-Zn accessions (IRIS_313-9368 and IRIS_313-10114), which had been identified in a screen of genebank accessions (Rakotondramanana et al., 2022). The four BF-Lines were selected based on their first-year grain Zn concentration, grain yield, and general phenotypic adaptation (i.e. no lodging, good panicle, and high number of tillers (data not shown)). In addition to these eight common entries, each location had a local farmer’s check variety, and the coastal sites had a fifth BF-Line (BF021) that had only shown promise in the coastal region in year 1.

### 2.3 Experimental layout and management

First-year trials were conducted in a Latin square design with two repetitions. Second-year agronomic trials used a split-plot design with three replications and factor mineral fertilizer application (NPK *vs.* no input) as the main factor and genotypes as the sub-factor. At all locations, each plot consisted of five 2-m-long rows. Thirty-day-old seedlings were transplanted as a single seedling per hill with 20-cm spacing between and within rows.

All experiments were conducted in farmers’ fields. NPK fertilizer (11:22:16) was applied at a rate of 300 kg/ha in all plots in the first year and at rates of 300 kg/ha at highland sites and 200 kg/ha at the coastal sites (in the NPK treatment only) in the second year. In all highland field trials, supplementary irrigation was provided from rainfed small creeks, whereas dry season experiments at the coastal sites were irrigated from a local reservoir.

At harvest, 21 plants within each plot were cut, and panicles were separated from straw and air-dried for a week in the laboratory. The weight of air-dried panicles was recorded to estimate grain yield (GY) in t/ha. For the determination of grain Zn concentrations, 10 panicles that were free of soil or other contaminants were sampled per plot and brought to the laboratory to be oven-dried for 3 days. Seeds of these panicles were dehulled manually to avoid metal contamination, and 2–5 g dehulled grain per sample was sent to Flinders University, Australia, for determination of [Zn].

At Flinders University, 0.3 g dehulled, brown, unbroken rice seed was oven-dried at 80°C for 4 h to remove remaining moisture, and the exact weight and grain number were recorded, followed by acid digestion in a closed tube as described in Wheal et al. (2011) (Wheal et al., 2011). Elemental concentrations of samples were measured using inductively coupled–plasma mass spectrometry (ICP-MS 8900; Agilent, Santa Clara, CA, USA) according to the method of Palmer et al. (2014) (Palmer et al., 2014). A blank and a certified reference material (CRM; NIST 1568b rice flour) were analyzed for every 30 samples for quality assurance. Samples with aluminum (Al) present at >5 μg/g were considered to have unacceptable levels of purported soil contamination (Yasmin et al., 2014); thus, they were eliminated from the dataset. Grain [Zn] is given in μg/g on a dry weight basis.

### 2.4 Statistical analysis

Within each trial, basic statistics were applied to validate the assumptions for variance analysis and discard potential outliers based on Cook’s distance. Linear mixed models were used to partition variance into the sources of variation defined by the experiment (Bates et al., 2015). For the year 1 trials conducted in a Latin square design, the following model was used:


(Model 1) $$y_{ijkrc}=\mu \;+\;g_{i}+s_{j}+gs_{ij}+r{\left(s\right)}_{jk}+row{\left(r\left(s\right)\right)}_{jkr}+col{\left(r\left(s\right)\right)}_{jkc}+\varepsilon_{ijkrc},$$

where *y_ijk_* is the phenotypic value of genotype *i* in site *j* evaluated in the *k*th rep, μ is the general mean of the experiment, *g_i_* is the genotypic value of *i*th genotype, $s_{j}$ is the effect of the *j*th site, *gs_ij_* is the interaction between the *i*th genotype with the *j*th site, *r_jk_* is the effect of the *k*th repetition nested in the site, *row_jkr_* and $col_{jkc}$ are the effect of the *r*th row and *c*th column both nested in the repetition nested in the site, respectively, and *ε_ijkrc_* is the residual term. Model selection with all the terms was performed with the stepwise algorithm based on the Akaike information criterion (step AIC), which chooses a model by AIC in a stepwise algorithm. Only the design factors (repetition, row, and column effects) were modeled as random effects.

For the year 2 trials, the split-plot design was modeled, as follows:


(Model 2) $$y_{ijkl}=\mu \;+\;g_{i}+s_{j}+t_{k}+gs_{ij}+gt_{ik}+st_{jk}+gst_{ijk}+r{\left(t\left(s\right)\right)}_{jkl}+\varepsilon_{ijkl}.$$

The main effects are the same as in Model 1, with an additional *t_k_* for the effect of the fertilizer treatment (*k* = 1, 2), *gs_ij_* is the interaction between the genotype and the treatment, *st_jk_* is the interaction between the site and the treatment, *gst_ijk_* is the interaction between the genotype the site and the treatment, *r_jkl_* is the effect of the *l*th repetition nested in the treatment in the site, and *ε_ijkl_* is the residual term. Similarly, the model was selected following a step AIC. Only the repetition factor was modeled as random effects, while all other model terms were fixed effects.

In the year 2 trials, fertilizer treatments were compared using t-tests and genotypes within the environment, and fertilizer treatments were compared with a pairwise test performed on lsmeans with Tukey’s method for pvalue adjustment. To compare genotypes against the baseline (X265) at each environment and fertilizer treatment, the multiple t-test was performed with the Dunnett’s test.

Within each year’s set of trials, the variance component estimates were obtained. For each factor considered as a fixed effect, the Eta^2^ was calculated as the ratio of the Sum of Squares (SSq) of the effect to the total SSq of all fixed effects. Trial-wise best linear unbiased estimated (BLUE) were obtained using the models per site and were used for the GEI studies and the graphical visualization of the crop performance. For the GGE plotting, two-way tables listing the adjusted mean value of the genotypes for each environment were used. The GGE biplots were performed from the first two principal components (Component 1 and Component 2) that were derived from subjecting environment-centered GY and grain [Zn] means for each environment (centering = “tester”) to singular value symmetrically partitioned into the entry and the environment eigenvectors (SVP = “symmetrical”) and with scaling of environment by standard deviation (scaling = “sd”).

Additional estimation of the genotypes’ stability was performed using the additive main effects and multiplicative interaction (AMMI) model. The stability of genotypes across all environments within a year trial or combined year trials was assessed using the AMMI stability value (ASV) coefficient calculated based on the AMMI models’ IPCA1 and IPCA2 (interaction principal component axes 1 and 2, respectively) scores for each genotype. The lower the ASV, the greater the stability of the genotype in the studied environments.

All statistics were performed using R 3.5.0 (R Core Team, 2023) and appropriate packages. The linear mixed models were analyzed with lme4 (Bates et al., 2015) CRAN package. The GEI studies were performed with gge (Wright and Laffont, 2021) and the plotting with Metan (Olivoto and Lúcio, 2020) CRAN packages. The AMMI was conducted with the agricolae (de Mendiburu, 2023) CRAN package.

## 3 Results

### 3.1 Baseline Zn concentration of local check varieties

**Figure 1:**
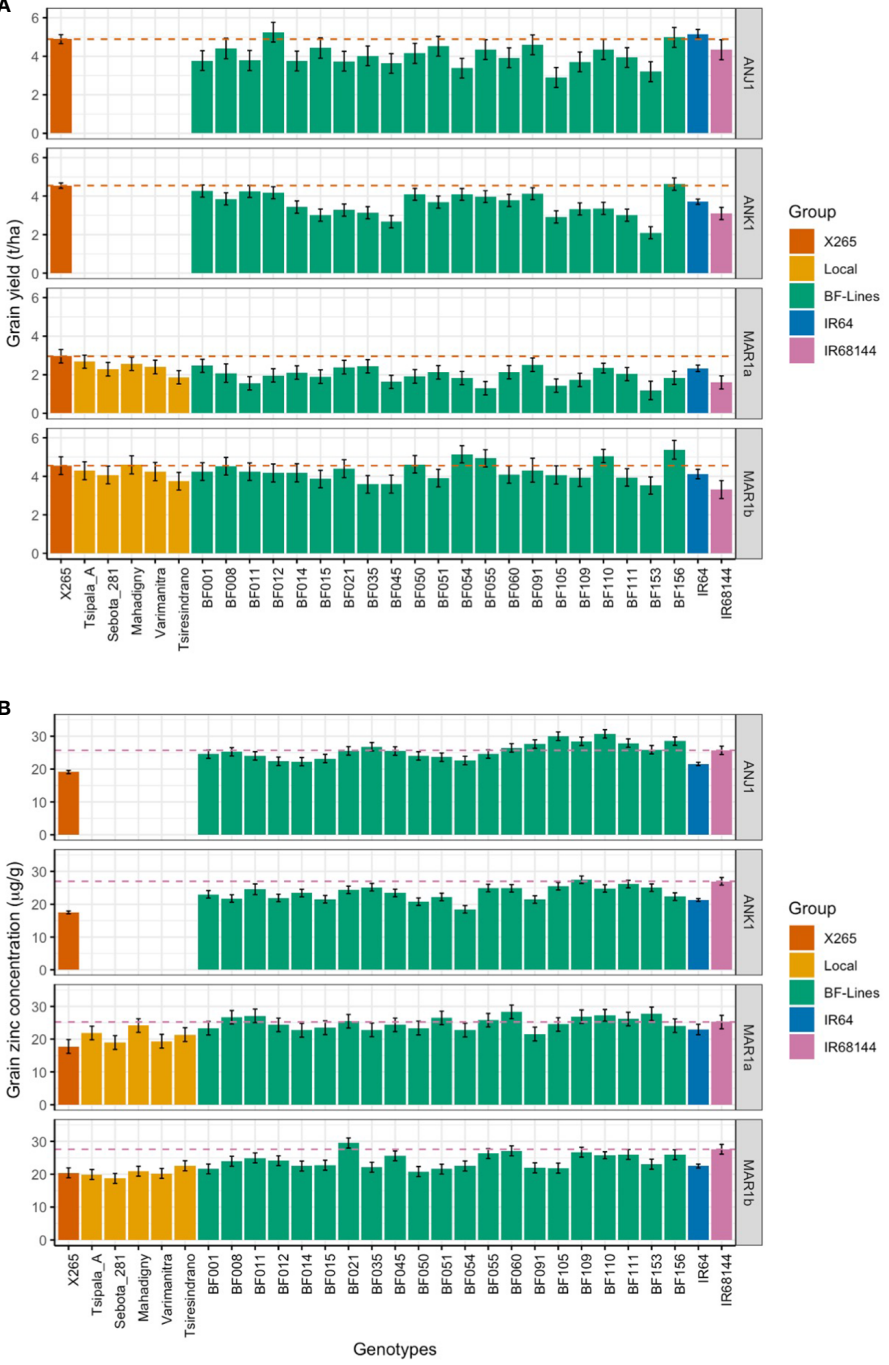
Grain yield (GY; **A**) and grain zinc concentrations (grain [Zn]; **B**) in year 1 trials of the national check X265, five farmers’ varieties, 21 BF-Lines, the mega-variety IR64, and the high-Zn check IR68144 at two sites located in the highlands (ANJ1 and ANK1) and two coastal sites (MAR1a and MAR2a). Dashed lines represent the performance of X265 and IR68144 for GY and ZN, respectively.

The recommended variety for the central highlands of Madagascar is X265, and its mean grain [Zn] ranged from 15.0 to 23.5 μg/g at Anjiro and Ankazomiriotra (**Table 3**), with an average across sites of 18.4 ± 1.8 μg/g. For the northwestern coastal region, no single recommended variety exists, and we therefore determined grain [Zn] for the five most popular varieties: Tsipala_A, Sebota_281, Mahadigny, Varimanitra, and Tsiresindrano. These local varieties ranged from 18.0 to 25.0 μg/g (**Table 3**, **Figure 1**) with an average of 20.8 ± 2.0 μg/g. Based on these analyses, we can define baseline [Zn] of 18.3 μg/g for the central highlands and 20.8 μg/g for the coastal region.

**Table 3:** Grain yield (GY) and grain zinc concentrations (grain [Zn]) of the five groups of genetic material utilized in experiments conducted in year 1 trials.

Group	GY (t/ha)				grain [Zn] (µg/g)			
	Range	Mean	sd	CV (%)	Range	Mean	sd	CV (%)
National check (X265)	2.54–6.22	4.63	0.78	16.81	15.0–23.5	18.39	1.81	9.82
Local check (5 lines) ^(a)^	1.50–5.70	3.25	1.12	34.53	18.0–25.0	20.85	2.03	9.75
BF-Lines (21 lines)	1.14–5.75	3.49	1.15	32.95	18.6–33.0	24.63	2.85	11.57
Mega-variety check (IR64)	1.19–6.25	3.88	1.21	31.18	20.0–27.0	22.04	1.65	7.50
High-Zn check (IR68114)	1.09–5.13	2.97	1.28	43.05	22.1–29.3	26.24	2.67	10.18

^(a)^ Data reported from MAR1a and MAR1b sites only.

### 3.2 Effects of genotype (G) and environment (E) on grain yield and grain Zn concentrations in year 1 trials

The effect of E differed considerably between GY and grain [Zn] (**Table 4**). While the factors G and E and the G by E interaction (GEI) contributed almost equally to the variance for GY, the variance for grain [Zn] was mainly due to variation between genotypes (76%) and the GEI (23%) effect. Average grain [Zn] was similar between environments (**Figure 1**, **Supplementary Table 1**) with a narrow range of 22.0 to 24.0 μg/g. In contrast, GY ranged from as low as 2.1 t/ha in MAR1a to 3.8 t/ha in ANK1, 4.2 t/ha in MAR1b, and 4.4 t/ha in ANJ1 (**Figure 1**, **Supplementary Table 1**). The range in GY could not be attributed to differences in soil properties, possibly because all sites were P deficient with Olsen-P values below 5 mg/kg, and soil pH was in a narrow range, pH of approximately 5.0 (**Table 1**).

**Table 4:** Variance components for grain yield (GY) and grain zinc concentrations (grain [Zn]) based on the analysis across locations within the year 1 trials resulting from the linear mixed model (Model 1).

	GY (t/ha)				grain [Zn] (µg/g)			
	df	MSq	Eta^2^	p value	df	MSq	Eta^2^	p value
Genotype (G)	28	1.09	37%	<0.001	28	47.39	76%	<0.001
Environment (E)	3	8.89	33%	<0.001	3	3.27	1%	0.443
GxE	74	0.33	30%	0.009	74	5.45	23%	0.001
Residual	90	0.20		<0.001	93	2.98		<0.001

Eta^2^ was calculated as the ratio of the Sum of Squares (SSq) of the effect to the total SSq of fixed effects.

The national check variety X265 had the highest or among the highest average GY in all four environments (**Figure 1**). Nevertheless, the range of GY varied notably, from as low as 3.2 t/ha in MAR1a to 4.9 t/ha in ANJ1. The group of 21 imported lines (BF-Lines) varied considerably across the sites. Half the BF-Lines had GY below 2 t/ha in low-yielding MAR1a, and several were equal to or exceeded X265, especially at the MAR1b site. IR68144 was used as a reference across environments for its relatively stable high grain [Zn], being the referential for HarvestPlus rice evaluation. Its grain yield was generally low, while grain [Zn] was indeed among the highest with an average of 26.2 μg/g compared to 22.0 μg/g for IR64 and only 18.4 μg/g for X265 (**Table 3**). BF-Lines had an average of 24.7 μg/g with best lines reaching 33 μg/g. Compared to the high zinc check, in all four environments, at least one BF-Line had superior grain [Zn], and in ANJ1 and MAR1a, nine and 10 surpassed the grain [Zn] observed in IR68144, respectively.

**Figure 2:**
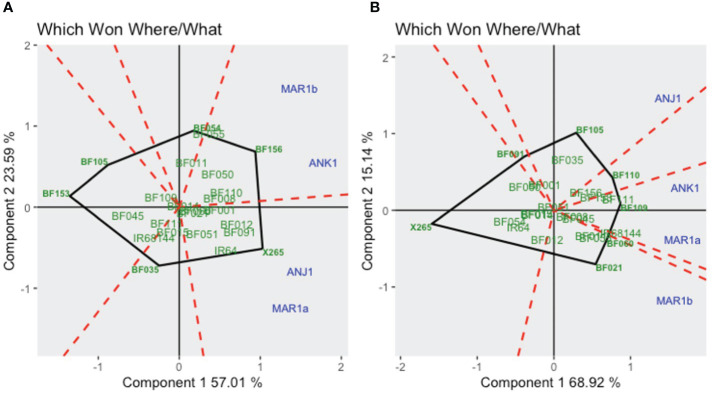
GGE biplot “*Which Won Where/What*” for grain yield **(A)** and grain zinc concentrations **(B)** for year 1 trials using symmetrical SVP and tested centered G+GE with scaling by standard deviation.

GGE biplots dissect the complex nature of GEI and simplify them into principal components (Component 1 and Component 2), which accounted for 57.0% and 23.6% of the total GGE variation for GY and 68.9% and 15.1% for grain [Zn], respectively (**Figure 2**). The *Which One Where/What* feature highlights the best genotype in a specific mega-environment as those plotted far from the biplot origin and closer to tested environments. For GY, X265 was “winning” in the MAR1a-ANJ1 mega-environment, and BF156 was the winner in ANK1-MAR1b (**Figure 2A**). For grain [Zn], the global grouping into mega-environments was less clear, possibly because GEI as captured by Component 2 (15.1%) was smaller than for GY (23.6%). BF109 won in the MAR1a-ANK1 mega-environment, whereas BF021 won in MAR1b and BF105 in ANJ1 (**Figure 2B**). It was interesting to note that national check variety X265 was placed by itself at the opposing end along Component 1.

**Figure 3:**
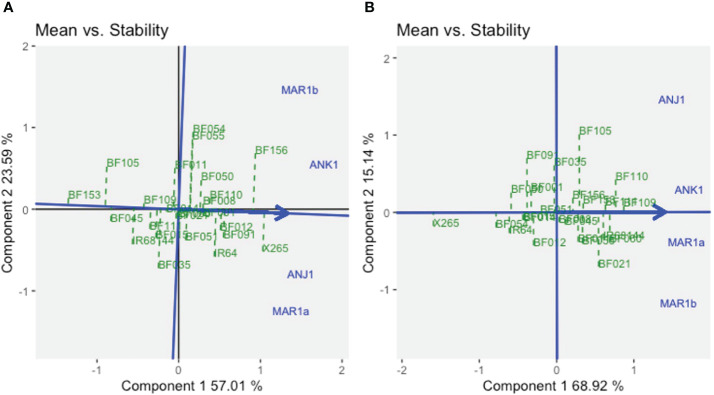
The GGE biplot “*Means vs. Stability*” for grain yield **(A)** and grain zinc concentration **(B)** for year 1 trials using symmetrical SVP and tested centered G+GE with scaling by standard deviation.

The stability of the genotypes could be assessed using graphs on *Mean vs. Stability* (**Figure 3**). The arrowhead line represents the average environment axis (AEA), and its length is a measure of the relative importance of the genotype main effect (G) *vs.* the GEI. The perpendicular deviation from the AEA indicates the stability of a genotype with small deviations signifying high stability. The best average performance for GY across all environments was found for X265 with moderate stability (ASV = 0.30), while BF156 was the second best but was less stable (ASV = 0.84) (**Figure 3A**, **Supplementary Table 2**). BF109 was the best line for grain [Zn], and this was coupled with the highest stability (ASV = 0.38), while the second best, BF110, was less stable (ASV = 1.60) (**Figure 3B**, **Supplementary Table 2**).

Based on this year 1 field evaluation, five BF-Lines (BF109, BF110, BF156, BF055, and BF021) were selected for further experiments in year 2. BF109 and BF110 were selected for their high average grain [Zn]. BF156 and BF055 were selected for their stable above-average grain [Zn] coupled with high GY in BF156 or high GY in the MAR1b environment for BF055. BF021 was selected for representing a certain amount of GEI, as it had high grain [Zn] only at MAR1b (**Supplementary Figure 2**). It was therefore only tested further in the coastal region. These BF-Lines were compared to two checks common across all sites and years (X265 and IR64) and to two genebank accessions “IRIS” previously identified as having high grain [Zn] in Madagascar (**Table 2**).

### 3.3 Effect of fertilizer treatment (T) on grain yield and grain [Zn] in year 2 trials

Experiments in year 2 were conducted in four locations and seven fields (**Table 1**) and differed from year 1 trials in as much as a zero-input treatment representing typical farmer’s practice was added. At each site, the effect of omitting the NPK fertilizer application on GY and grain [Zn] could thus be evaluated. GY was most strongly affected by the factor E (35%), followed by G:E (21%), G (14%), and T (12%) effects (**Table 5**). In contrast, grain [Zn] was predominantly affected by variation linked to G (83%), whereas G:E or E effects were small, 9% and 4%, respectively, and the T effect was not detected.

**Table 5:** Variance components for grain yield (GY) and grain zinc concentrations (grain [Zn]) based on the analysis across locations within the year 2 trials using the linear mixed model (Model 2).

	GY (t/ha)				grain [Zn] (µg/g)			
	df	MSq	Eta^2^	p value	df	MSq	Eta^2^	p value
Genotype (G)	8	4.39	14%	<0.001	8	1733.84	83%	<0.001
Environment (E)	6	14.90	35%	<0.001	6	109.62	4%	<0.001
Treatment (T)	1	31.17	12%	<0.001	1	10.62	0%	0.162
GxE	46	1.16	21%	<0.001	48	31.04	9%	<0.001
GxT	8	0.85	3%	0.008	8	12.60	1%	0.015
ExT	6	3.77	9%	<0.001	6	10.57	0%	0.092
GxExT	45	0.34	6%	0.368	48	9.25	3%	0.003
Residual	198	0.32			217	5.16		

Eta^2^ was calculated as the ratio of the Sum of Squares (SSq) of the effect to the total SSq of fixed effects.

Fertilization with NPK affected GY significantly (p< 0.1 or p< 0.05) and positively in four out of seven environments (**Supplementary Figure 3A**, **Supplementary Table 3**). Average grain yield without NPK fertilizer ranged from as low as 1.1 ± 0.4 t/ha at BEN2b to 3.5 ± 0.6 t/ha at ANJ2a, and the range obtained with NPK fertilizer applications was from 1.9 ± 0.6 t/ha at ANK2 to 5.3 ± 0.8 t/ha at MAR2b. The most pronounced response to NPK was detected at BEN2a, where GY nearly tripled from 1.2 to 3.3 t/ha. An effect of NPK fertilization on grain [Zn] was not detected in any environment (**Supplementary Figure 3B**, **Supplementary Table 3**).

**Figure 4:**
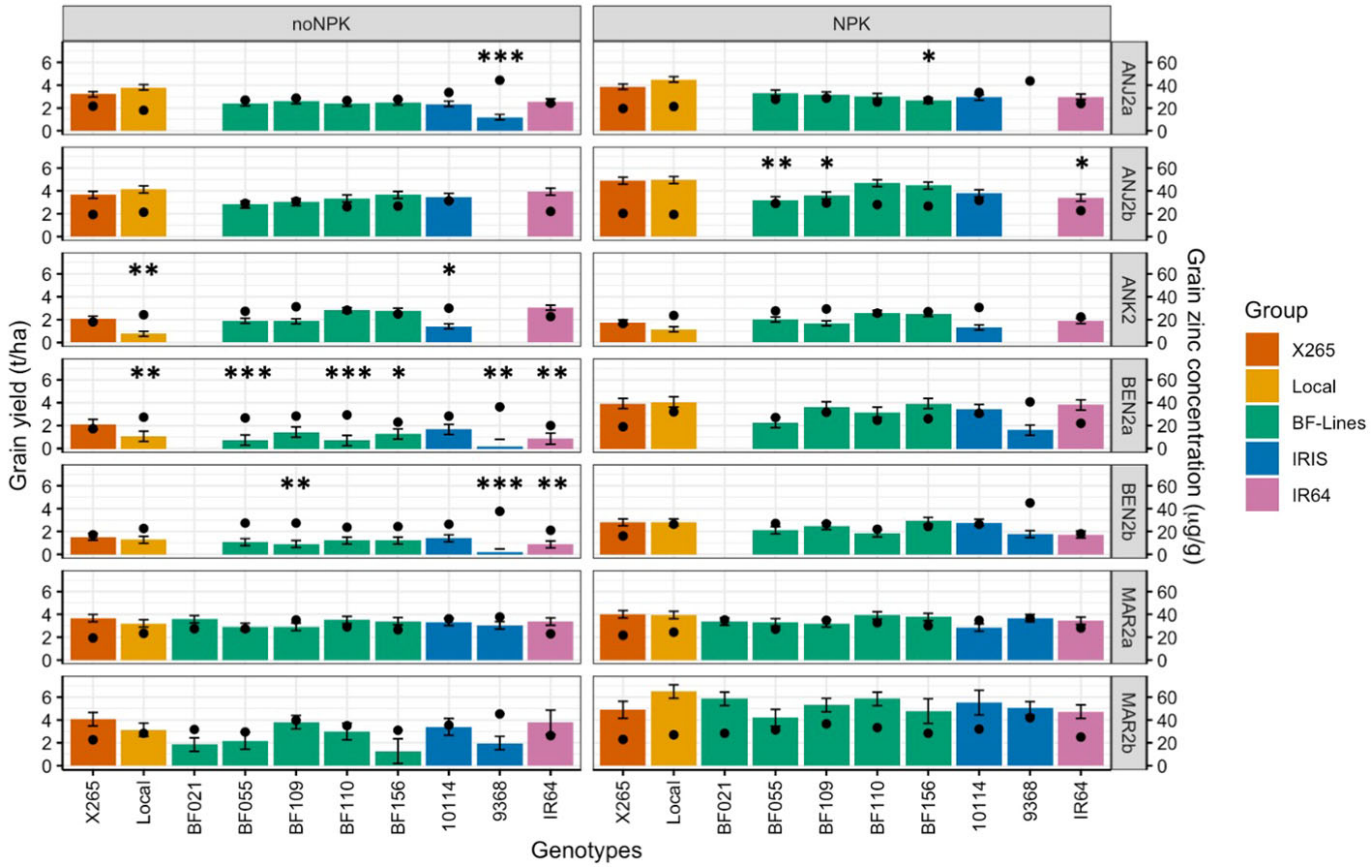
Grain yield (GY) and grain zinc concentrations (grain [Zn]) of the national check (X265), the local farmer’s varieties (Local), five Zn-biofortified lines (BF-Lines), two genebank accessions (IRIS), and the mega-variety (IR64) in five highland sites (ANJ2a, ANJ2b, ANK2, BEN2a, and BNE2b) and two coastal sites (MAR2a and MAR2b) in 2020. Average grain [Zn] is represented by black dots and average GY by colored bars with error bars for the standard deviation. The experiment was conducted in seven environments under two fertilizer treatments: zero input “noNPK” and supply of NPK “NPK”. Mean GY of lines was compared to check X265 through Dunnett’s test. Significant differences are indicated by ***, **, and * for p< 0.001, 0.01, and 0.05, respectively.

While the average effects of NPK on grain [Zn] were not significant, it is of interest to investigate whether higher GY in the NPK treatment led to a dilution of grain [Zn]. In the highly NPK-responsive site BEN2a, GY of X265 almost doubled from 2.1 to 3.9 t/ha, but grain [Zn] remained relatively constant at 17 and 19 μg/g, and very similar values were observed at all other environments (**Figure 4**, **Supplementary Table 4**). The same pattern was observed for high-[Zn] genebank accession IRIS_313-10114 that doubled GY in BEN2a (1.7 to 3.4 t/ha) and marginally increased grain [Zn] from 28.3 to 30.7 μg/g under NPK. Even at the highest yielding site MAR2b with GY between 4.2 and 6.1 t/ha in the NPK treatment, no consistent decrease in grain [Zn] was observed. The genebank accession IRIS_313-9368 more than doubled GY from 2.0 to 5.0 t/ha in response to NPK in MAR2b, but its exceptionally high grain [Zn] remained above 42 μg/g (**Figure 4**).

IRIS_313-9368 frequently had missing or extremely low values for GY due to its very early maturity, which rendered it prone to attacks by rats in the highland environments, as it was frequently the first rice to mature in the entire village. Despite the missing GY, enough panicles could be harvested to obtain an estimate of grain [Zn], which was the highest of all genotypes tested with a global mean of 40.9 ± 3.6 μg/g and a range of 36.3 to 51.7 μg/g across environments and treatments (**Figure 4**). Also, with a very narrow range but at the opposite spectrum for grain [Zn] with a mean of 19.4 ± 2.2 μg/g was X265, which had the lowest grain [Zn] in six of the seven environments. Among BF-Lines, the highest average grain [Zn] was detected in BF109 (31.3 μg/g), and this was achieved with a GY that was generally not significantly (Tukey alpha = 0.05) different from X265, except in BEN2a without NPK and ANJ2b with NPK, where BF109 yielded significantly (Tukey alpha = 0.05) lower GY. National check X265 had either the same or significantly (Tukey alpha = 0.05) higher GY compared to local farmer varieties (“Local”) (**Figure 4**, **Supplementary Table 4**).

**Figure 5:**
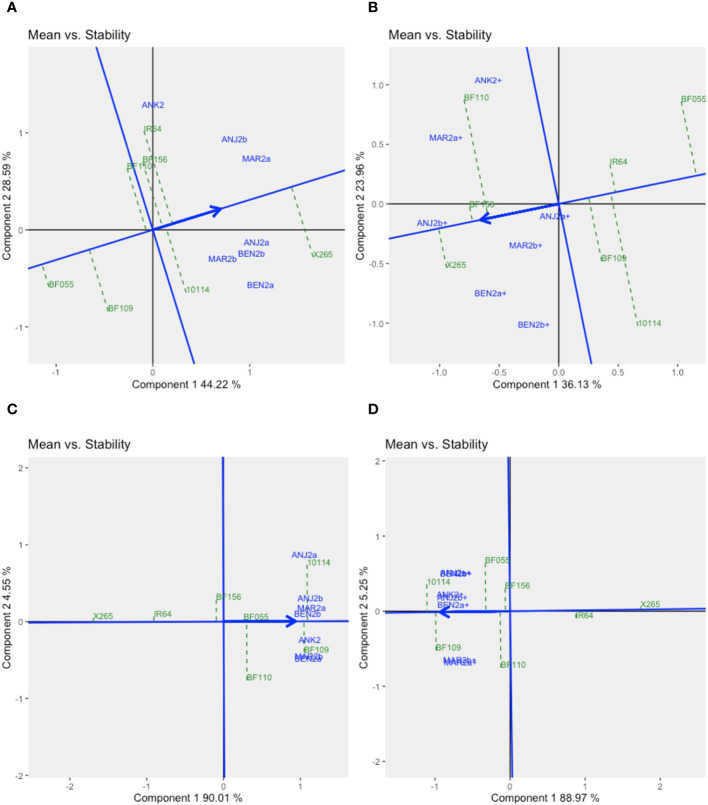
GGE biplots “*Means vs. Stability*” for year 2 trials in seven environments for grain yield under zero input **(A)** or NPK fertilizer **(B)** addition and for grain zinc concentration under zero input **(C)** or NPK fertilizer **(D)** addition. The GGE biplots were created based on symmetrical SVP and tested centered G+GE with scaling by standard deviation.

GGE biplots for year 2 trials were prepared separately for fertilized and unfertilized environments (**Supplementary Figures 4, 5**). For GY, Component 1 explained 44% and 36% of the GGE variation under no-NPK and NPK environments, respectively. Component 2, representing the GEI effect, accounted for 29% and 24% of the variation. With no additional fertilization, environments were grouped into three mega-environments, one being defined by ANJ2b and MAR2a, the highly productive sites non-responsive to fertilizer application (**Supplementary Figure 3A**, **Supplementary Table 2**). Another was defined by one site, ANK2, as relatively average in terms of productivity and also non-responsive to NPK. The remaining four sites, characterized by moderate-to-low productivity and NPK-responsive environments, formed the third mega-environment. Under NPK treatment, only the NPK-responsive environments remained relatively grouped together (as traduced by the angle between the vectors of environments approximated by the biplot), although not significantly (p< 0.05) correlated (**Supplementary Figure 5A**). The *Mean vs. Stability* GGE biplot (**Figure 5**) revealed that X265 had the highest average yield across all environments and was more stable in the NPK treatment than under no-NPK (ASV = 0.48 and 1.3, under NPK and no-NPK treatments, respectively, **Supplementary Table 2**). However, other genotypes showed superior average yield specifically in the ANK2 site, which differed according to the fertilizer treatment, and those were IR64 and BF110, under no-NPK and NPK treatments, respectively. Interestingly, BF156, which was the most unstable genotype under no-NPK treatment (ASV = 2.68), showed high stability (ASV = 0.06) across all sites under NPK treatment as well as a good average yield (GY = 3.57 t/ha).

For grain [Zn], G effects along Component 1 explained approximately 90% of the variation, and GEI effects were therefore small (approximately 5% for Component 2), causing all seven environments to cluster rather closely together (**Supplementary Figure 4C, D**). With little differences between environments for grain [Zn], we investigated to what extent genotypic means were stable across environments. X265 and mega-variety IR64 were very stable and thus did not vary in their low grain [Zn] depending on the environment and regardless of the fertilization treatment (**Figure 5**, **Supplementary Table 2**). Of the BF-Lines, BF156 had an average and relatively stable grain [Zn] across environments (ASV = 1.04 and 0.94 under conditions without and with NPK, respectively). BF110 was among the least stable (ASV = 2.59 and 2.19 under conditions without and with NPK, respectively), which was due to a higher frequency of intermediate grain [Zn] levels (**Figure 4**, **Supplementary Table 4**). The overall higher grain [Zn] of BF109 with more than 31 μg/g Zn in both treatments came with some degree of instability (ASV = 3.43 and 1.69 in the treatment without and with NPK, respectively).

**Figure 6:**
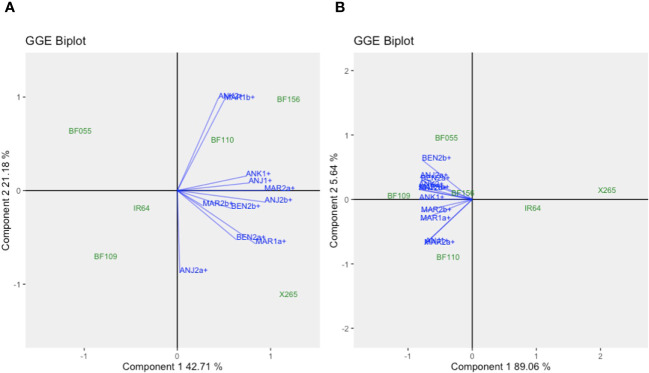
GGE biplots for the across-year analysis for grain yield **(A)** and grain zinc concentration **(B)** of check varieties IR64 and X265 and the four biofortified lines (BF-Lines) evaluated with NPK fertilization in year 1 and 2 trials. The GGE biplots were created using symmetrical SVP and tested centered G+GE with scaling by standard deviation.

A core set of four BF-Lines together with X265 and IR64 had been repeated at all sites in both years and were used for a further GEI analysis across years and sites only considering the environments with NPK treatment. For GY environments clustered independent of the year or geographical proximity (**Figure 6**; **Supplementary Table 1**), for example, MAR1b and ANK2 behaved similarly, and BF156 and BF110 appeared adapted to these environments. In a different group, MAR1a and BEN2a were highly similar, and X265 was the most adapted. As for individual years, the across-year analysis for grain [Zn] indicated that factor G on Component 1 was dominant, explaining approximately 89% of the variation, while E and GEI effects only accounted for approximately 6% (**Figure 6**). Unlike for GY, BF-Lines clearly separated from both checks along Component 1, and BF109 had the highest overall grain [Zn] without being associated with a specific group of environments, while BF156 was average (26.4 μg/g Zn) and stable across environments (ASV = 0.89) (**Supplementary Table 2**).

## 4 Discussion

### 4.1 Baseline grain [Zn] and breeding targets for Madagascar

African countries like Sierra Leone, Guinea, Madagascar, and Liberia have a high-Zn biofortification priority index (BPI) based on the rate of per capita rice consumption, country rice production, and the prevalence of Zn deficiencies in the country (Broadley et al., 2007). However, to our knowledge, only one study reported on grain Zn concentrations in African varieties, showing a range of 16 to 27 μg/g Zn in dehulled brown rice of 26 Malagasy local varieties with a mean of 24 μg/g (Rakotondramanana et al., 2022). Among the varieties more commonly found in markets of towns and cities, Tsipala and Makalioka contained 20 and 21 μg/g, respectively.

One objective of our study was to provide more basic data on grain [Zn] across varieties and environments of Madagascar. In the central highlands where much of the rice is produced and consumed, the recommended and frequently grown variety is X265. Based on our data from four regions and two years, we can establish that X265 is consistently among the varieties with low grain [Zn], averaging just 18.4 μg/g Zn in dehulled brown rice. We therefore consider 18 μg/g to be the baseline for brown rice Zn in the central highlands where rice is grown during the rainy season. The coastal region of Madagascar has a much warmer climate, and trials were conducted during the dry season, relying on irrigation. Different varieties from highland regions are cultivated, of which the five most popular were tested here. They ranged from 18 to 25 μg/g in brown rice with an average of 20.9 μg/g, which one may consider the baseline for the coastal regions during the dry season. X265 averaged 19.0 μg/g in that region. The slightly higher grain [Zn] in the coastal area may be due to a typical seasonal effect: grain [Zn] tends to be higher in the dry season compared to wet season trials (Goloran et al., 2019; Suman et al., 2021).

Polishing brown rice further reduces grain [Zn] by 15% to 20% on average (Taleon et al., 2020). We can thus anticipate that our Malagasy baseline of 18 and 21 μg/g estimated for brown rice would correspond to 15 to 17 μg/g Zn in polished white rice, which is comparable to the grain [Zn] baseline of 16 μg/g Zn assumed for rice generally (Bouis and Welch, 2010). Based on this typical baseline, the long-term breeding target for Zn biofortification in rice established by HarvestPlus is an improvement of [Zn] to 28 μg/g in polished white rice (Virk et al., 2021). The BPI established by HarvestPlus assumes that such a level would deliver sufficient Zn *via* rice to alleviate Zn malnutrition in populations relying on rice as their primary food source (https://bpi.harvestplus.org). However, it is recognized that this long-term goal of +12 μg/g Zn may require several breeding cycles to be achieved, and a short-term goal of reaching at least 50% of target concentrations has therefore been suggested during the initial stage of biofortification breeding (Virk et al., 2021). Referring to brown rice, our short-term breeding target would thus be approximately 25 μg/g for the highlands and 27 μg/g for the coastal region.

### 4.2 Levels of grain [Zn] in local varieties and breeding lines

Of the 35 breeding lines and varieties tested, 12 were popularly grown local varieties, and none of these consistently reached this breeding target. Neither did the 26 accessions tested previously (Rakotondramanana et al., 2022). While many more local varieties exist *in situ* and in genebanks that should be evaluated for grain [Zn], we cautiously conclude that reaching breeding targets likely requires outside genetic resources such as the 20 BF breeding lines imported from CIAT. Of these, several reached target grain [Zn], but only five combined this feature with acceptable grain yield and were selected for second-year trials that included additional farmer’s fields and a zero-input treatment representing farmers’ practice in the region.

Two genotypes, BF110 and BF156, were identified as having good grain yield across both years (average of 3.7 and 3.8 t/ha in the NPK treatment, respectively), which was not different from the yield of 3.9 t/ha of X265. Their grain [Zn] was above the breeding target (>25 μg/g). Even higher grain [Zn] was detected in BF109 (29.7 μg/g), but grain yield was lower at 3.3 t/ha (across years with NPK). BF110 and BF156 represent a good compromise between productivity and biofortification and were subsequently considered for variety release. Despite meeting grain yield and grain [Zn] targets, their poor panicle exertion (data not shown) prevented them from being accepted as the first Zn-biofortified varieties in Madagascar. This undesirable trait is often associated with increased susceptibility of the panicle to diseases that can negatively affect grain yields (Cruz et al., 2008). However, poor panicle exertion had not been observed in field trials in Latin America, ruling out a genetic origin. Other factors such as high temperatures occurring at the booting stage could be suggested (Cruz et al., 2008; Chen et al., 2020), but our understanding of this problem is still insufficient and requires further studies. While this problem prevents the utilization of BF110 and BF156 as varieties, a potential future for this germplasm would be to integrate them as parents in a breeding program of new biofortified rice and to test them further in other African locations. As such, other potential donors for the high grain [Zn] trait are BF109 and the two genebank accessions confirmed to have superior grain [Zn] in year 2 trials.

### 4.3 Stability of grain [Zn] across environments, years, and treatments

In a study of variations for grain [Zn] across soil types differing in Zn bioavailability, (Wissuwa et al., 2008) concluded that grain [Zn] can vary more than twofold (from below 10 to approximately 20 mg/g in the standard lowland rice variety IR72) depending on the soil-Zn status. Similar results were reported in a study comparing 68 genotypes across 15 locations in India, where close to twofold variation in grain [Zn] was detected between site means, and this variation could mainly be attributed to contrasting levels of plant-available Zn in the soil (Naik et al., 2020). In contrast, the effects of environments tested in our study were minor, and considering that [Zn] were comparable to the upper range found in the above studies, one may conclude that soil-Zn availability was not a limiting factor at any of our sites. This was expected given the acidic soil pH ranging from pH 4.7 to 5.5, which is within the range of optimal Zn availability. In both years, variation in grain [Zn] was mostly due to G effects (more than 76%), and GEI effects were comparatively small. Similar results were observed in studies where more genotypes were considered. Strong G effects were reported by (Babu et al., 2020), who screened a collection of 40 landraces and released varieties in three environments and attributed 66.7% of the variation to G compared to 27.4% for GEI effects. Larger G than GEI effects were also detected in screening 68 traditional genotypes and advanced breeding lines in multi-location trials over five provinces in India (Naik et al., 2020).

Contrasting to this pattern of dominant G effects, Suman et al. (2021) reported that only a very limited part of the variation for grain [Zn] contributed to G effects (8.1%) in their evaluation of 44 recombinant inbred lines (RILs) tested in four environments. Nearly all the variation was explained by the dominant E effect (89%) with GEI explaining only 1.9%. Two factors are likely responsible. The dominant E effect was caused by large seasonal effects with dry season grain [Zn] being 50% higher than in the wet season. However, more importantly, the small G effect may be explained by the limited diversity present in a bi-parental population. From a breeding perspective, this is of relevance because breeding populations tend to be less genetically diverse compared to collections of landraces used by Babu et al. (2020). The genetic diversity in the present study would be in between these extremes, with more diversity in year 2 brought by the inclusion of genebank material. Low estimates of GEI obtained in our study may therefore have to be considered in relation to this genetic diversity, and it is likely that the low GEI in year 2 (9%) is an underestimation and that year 1 estimates of 23% are closer to what may be expected in a true-breeding population.

In contrast to results for grain [Zn], GY was much more strongly affected by E and GEI effects. Considering the year 1 trials, GGE plots indicated the presence of two mega-environments that strongly affected yield levels generally but also in a genotype-specific way with strong GEI encountered in both years (**Figure 2A**). MAR1a and ANJ1 represented one mega-environment with the common feature of sufficient water supply through irrigation, which would explain the similar ranking of genotypes. Infrequent rains at the rainfed lowland ANK1 site and periodic salinity in coastal MAR1b may have contributed to a different ranking of the genotypes in the second mega-environment. For example, BF156 was the top line for GY at ANK1 and MAR1b, but its GY was below average at MAR1a. In year 2 trials, the comparison of a zero-input treatment (farmer’s practice) to NPK fertilizer application was included, and NPK application improved GY by an average of 69% across sites. A different grouping of environments was encountered depending on the NPK application, yet the NPK-responsive environments remained grouped together, with X265 being the winning genotype. Among the non-NPK-responsive environments, the site with average yield performance, ANK2, had a different ranking of the genotypes according to the soil fertilization scheme, with IR64 as the winning genotype in non-fertilized soils and BF110 when NPK was added (**Figures 5A, B**). This draws attention to the difficulty of predicting the best-performing genotype in such an environment when changing crop management options. The combination of the two year trials, only considering the NPK treatments, brought more complexity in the definition of mega-environments for GY, with change in the previously reported tendencies (**Figure 6**). Climatic conditions are factors of high relevance for the yield performance of genotypes evaluated in our selected sites and thus for the definition of mega-environments. Nevertheless, considering the four BF-Lines and the two checks evaluated across years and sites, BF156 remained among the highest yielders and most stable genotypes.

The significant GY enhancement under NPK at most sites except ANK was most likely due to the effect of P, as Malagasy soils are typically deficient of P (Rabeharisoa et al., 2012), which our soil analysis confirmed for all fields with an Olsen-P below 5 mg/kg. Negative correlations have often been reported between grain yield and grain [Zn] (Inabangan-Asilo et al., 2019), suggesting higher yields could have led to a dilution of Zn in the larger grain biomass. However, this effect was not observed here considering within-line or global variation for grain [Zn] relative to the variation for GY (**Supplementary Figure 6**). Even where GY doubled in response to NPK application, grain [Zn] remained constantly high (IRIS_313-9368; BF021) (**Supplementary Figure 3**). Furthermore, antagonistic effects between P and Zn have been reported, with high doses of P fertilizers potentially leading to an imbalanced P:Zn ratio in the soil that can reduce Zn uptake by the plant (Olsen, 1972). These effects were apparently of little importance here, possibly because the native soil-P levels are expected to be in the deficiency range (Rabeharisoa et al., 2012).

### 4.4 Breeding Zn-biofortified rice for Madagascar

Our 2-year study established that tested local varieties failed to reach Zn biofortification targets and that the recommended variety for Malagasy central highlands, X265, despite the highest grain yields, also had low grain [Zn]. Among the biofortified breeding lines imported from CIAT, several combined grain [Zn] levels at or above short-term breeding targets with acceptable GY and were thus candidates to enter further variety testing. However, their poor panicle exertion prevented a release. Nevertheless, based on the data generated, a likely breeding strategy emerges. Local varieties with good adaptation and yield potential would have to be crossed with donors of the high grain [Zn] trait. For this purpose, BF109, BF110, BF156, and BF021 would indeed be good candidate donors. Even higher grain [Zn] above 40 μg/g was consistently detected in genebank accession IRIS_313-9368, which belonged to the *aus* sub-group of rice that contains many other high-Zn accessions as potential donors (Rakotondramanana et al., 2022). This *aus* sub-group is genetically distant from the predominantly *indica*-type rice grown in the lowlands of Madagascar. This genetic distance may require a breeding scheme involving further backcrossing to recover the characteristics of *indica* rice.

Stable performance in grain yield and grain mineral concentration is essential for the successful development and diffusion of biofortified rice (Inabangan-Asilo et al., 2019). In order to combine high grain [Zn] with good grain yield, both traits need to be selected, and the difference in importance of GEI for grain [Zn] and GY poses the question of how this may best be achieved. The dominant G and smaller GEI effects for grain [Zn] indicate that selection for grain [Zn] could be centralized or limited to few sites. Based on GGE biplots for grain [Zn], no distinct mega-environments emerged, but several environments were grouped near the AEA and could thus be sites to conduct grain [Zn] evaluations. Correlations for grain [Zn] between 13 environments (**Supplementary Figure 5B**) indicated ANK2+ to be a rather representative environment, having strong correlations with eight of the 12 environments and may thus be suitable as a selection site. Similarly, MAR2b was highly correlated to other environments, and being located in the coastal region would allow for selections during the off-season, thereby speeding up the variety development process. Field evaluations of the most promising advanced generations prior to variety release could then be scattered in various farmers’ fields across environments in order to collect data relevant to the farmers’ field management effects.

For GY, distinct mega-environments were detected, and contrary to expectations, these did not distinguish highlands from coastal sites. Instead, higher-yielding environments represented by MAR2a and to a lesser extent by ANJ2b were distinct from environments partly characterized by the presence of abiotic stresses such as low soil fertility, salinity, or mild drought (ANJ2a, BEN2a, BEN2b, and MAR2b). The early-stage evaluation of large numbers of entries for GY should thus be conducted at least at one site per mega-environment. If resources are available and if breeding for adaptation to certain stresses is of importance, additional sites and evaluation years will be needed, and to optimize this process, sites should be characterized in more detail with regard to prevailing yield-limiting factors.

## 5 Conclusions

Low Zn concentrations found in grains of the most commonly grown local rice varieties could partly explain the high prevalence of Zn deficiency in Madagascar. Zn biofortification breeding should therefore be a priority and needs to rely on genetic resources such as the donors identified in this study, namely, four of the CIAT biofortified breeding lines (BF109, BF110, BF156, and BF021) and the *aus* accession (IRIS_313-9368). While BF156 had generally stable grain [Zn] reaching our breeding target, and relatively high and stable GY among sites with NPK application, BF109 had average and stable GY and superior yet less stable grain [Zn]. That genetic effects were consistent and threefold stronger compared to GEI effects facilitates breeding efforts. A concerted effort should be undertaken to further test high-Zn breeding lines developed elsewhere (Asia and Latin America) in Madagascar and other African target countries for Zn biofortification in rice. Similarly, the high-Zn lines and donors identified in this study, mostly those showing good stability such as BF156, are anticipated to exhibit elevated grain [Zn] elsewhere, making them potentially valuable for sharing with stakeholders across Sub-Saharan Africa. This could include considering an expedited release as the initial zinc-biofortified varieties in Africa, provided they demonstrate general adaptability and possess acceptable plant and grain characteristics.

## Data availability statement

The original contributions presented in the study are included in the article/**Supplementary Material**. Further inquiries can be directed to the corresponding authors.

## Author contributions

MR: Data curation, Formal analysis, Methodology, Writing – original draft. MW: Conceptualization, Investigation, Methodology, Writing – original draft. LR: Supervision, Writing – review & editing. TR: Supervision, Writing – review & editing. JS: Methodology, Writing – review & editing. CG: Conceptualization, Data curation, Formal analysis, Funding acquisition, Writing – original draft.
