# Supplementary material for: Stability of grain zinc concentrations across lowland rice environments favors zinc biofortification breeding
Source: Front Plant Sci. 2024 Feb 13;15:1293831. doi: 10.3389/fpls.2024.1293831 (PMC10896981; doi:10.3389/fpls.2024.1293831)
Supplement: Supplementary file 1 [file DataSheet_1.pdf]

Supplementary Material

Stability of grain zinc concentrations across lowland rice environments favors zinc biofortification breeding.

Mbolatantely Rakotondramanana, Matthias Wissuwa*, Landiarimisa Ramanankaja, Tantely Razafimbelo, James Stangoulis, Cécile Grenier*

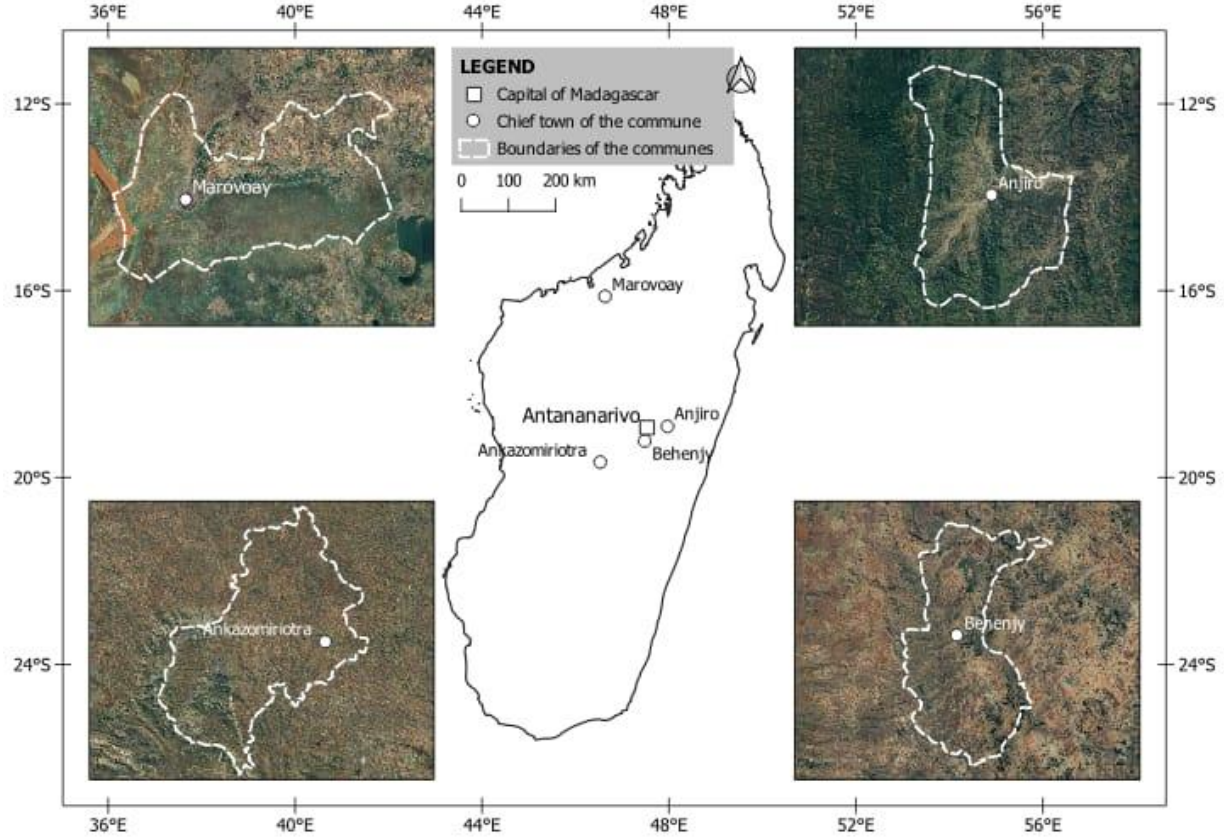

Supplementary Figure 1. Map of the sites considered in this study covering four regions of Madagascar; Marovoay in the coastal area, Anjiro, Ankazomiriotra and Behenji in the highlands. Map created using the Free and Open Source QGIS.

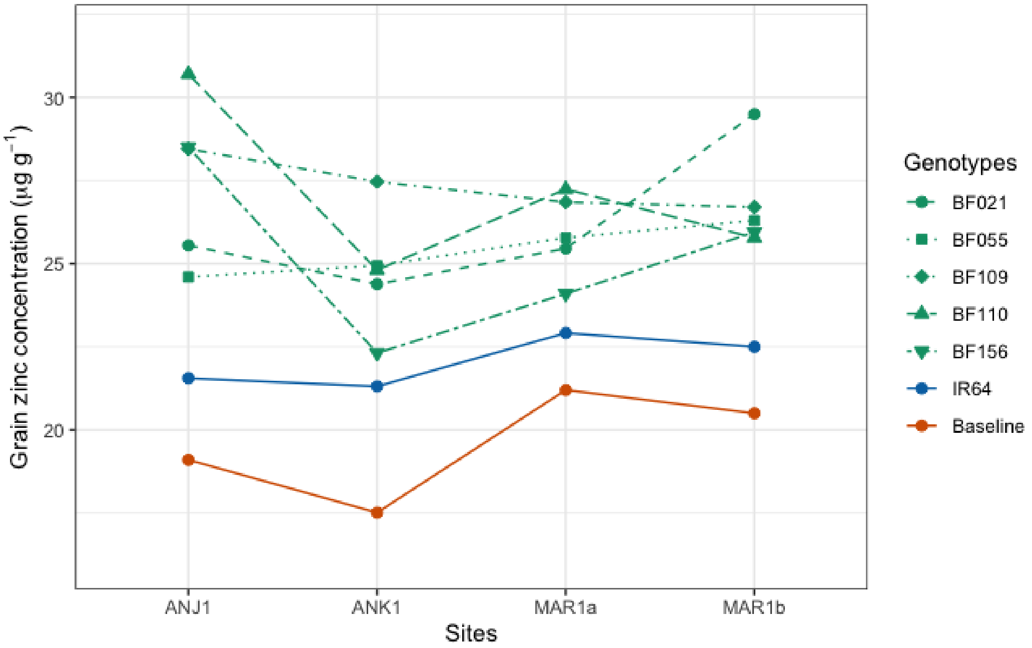

Supplementary Figure 2. Grain zinc concentration variation of the five selected biofortified lines (BF-Lines), the check IR64 and the baseline at four environments in year 1 trials, with ANJ1 and ANK1 in the highland and MAR1a and MAR1b in the coastal area.

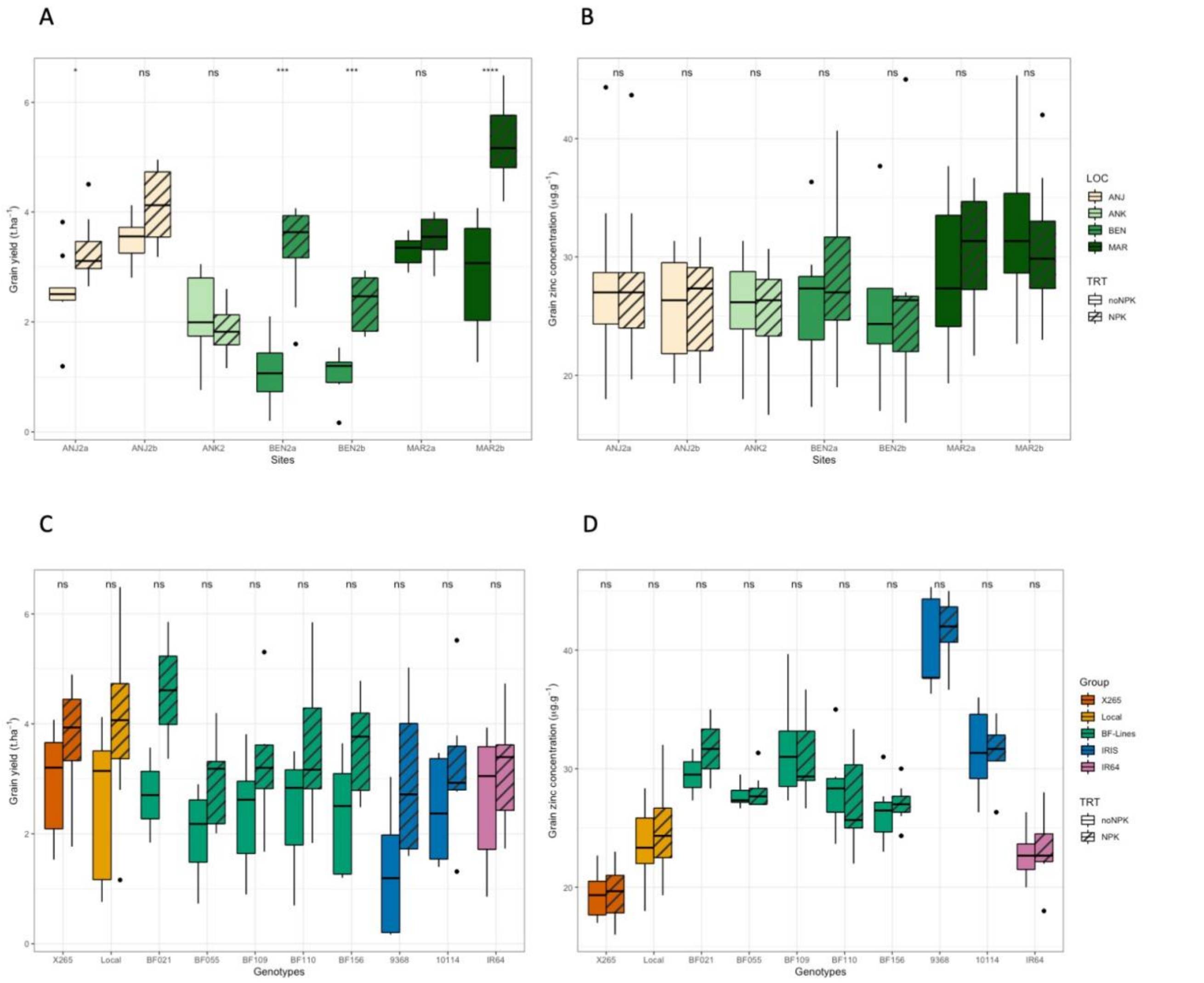

Supplementary Figure 3. Effect of NPK fertilizer on grain yield (A, C) and grain zinc concentration (B, D) grouping by environments (A, B) and by genotypes (C, D) all observations collected in the year 2 experiments. Colors in (A) and (B) correspond to the environments while colors in (C) and (D) correspond to the genotypes groups. Dashed boxes are for fertilized fields. Means between treatment were compared and *, ***, **** indicate significant difference at p<0.1, 0.01, and 0.001, respectively.

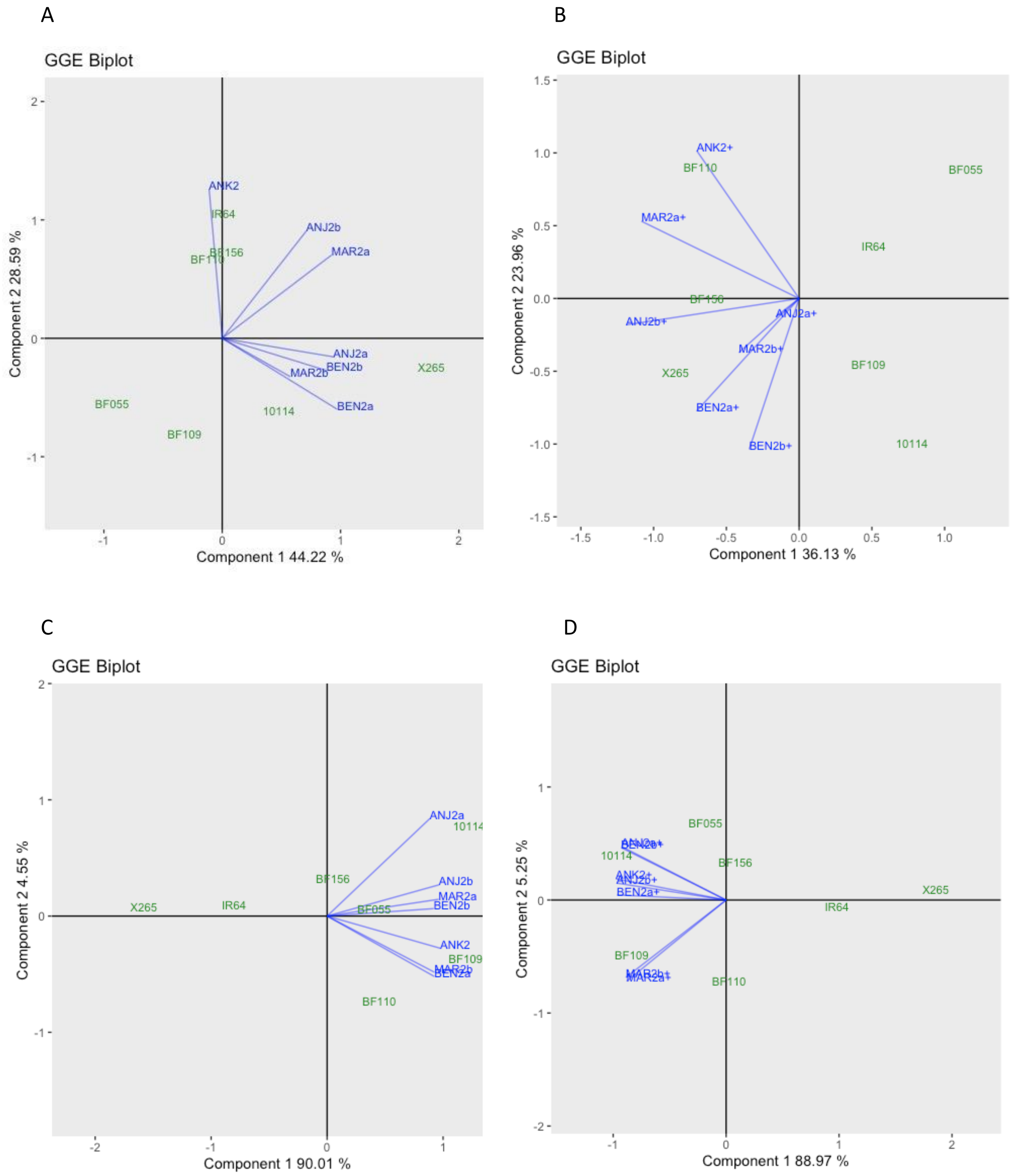

Supplementary Figure 4. The GGE biplot of GY for genotype comparison on seven environments under zero input (A) and NPK fertilizer (B) treatments, and grain zinc concentration under zero input (C) and NPK fertilizer (D) treatments during year 2 trials. The GGE biplots uses components 1 and 2 to explain 72.81% (A), 60.09% (B), 94.56% (C) and 94.22% (D) of the total variation. The biplots were created based symmetrical SVP and tested centered G+GE with scaling by standard deviation.

A

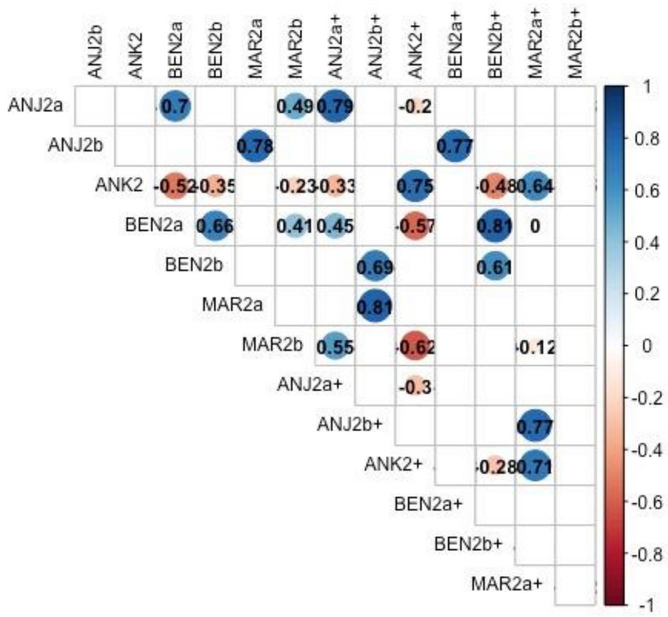

B

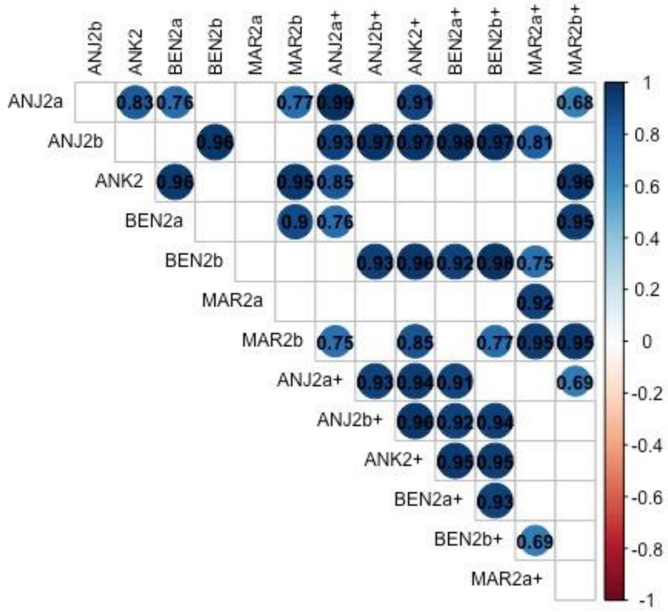

Supplementary Figure 5. Correlation (n = 27-30) between seven environments under no-NPK and NPK (with the added "+" sign) treatment observed for grain yield (A) and grain zinc concentration (B). The environments were distributed into highlands sites (ANJ2, ANK2 and BEN2) and the sites in the coastal area (MAR2). Only correlations with $p<0.05$ are shown.

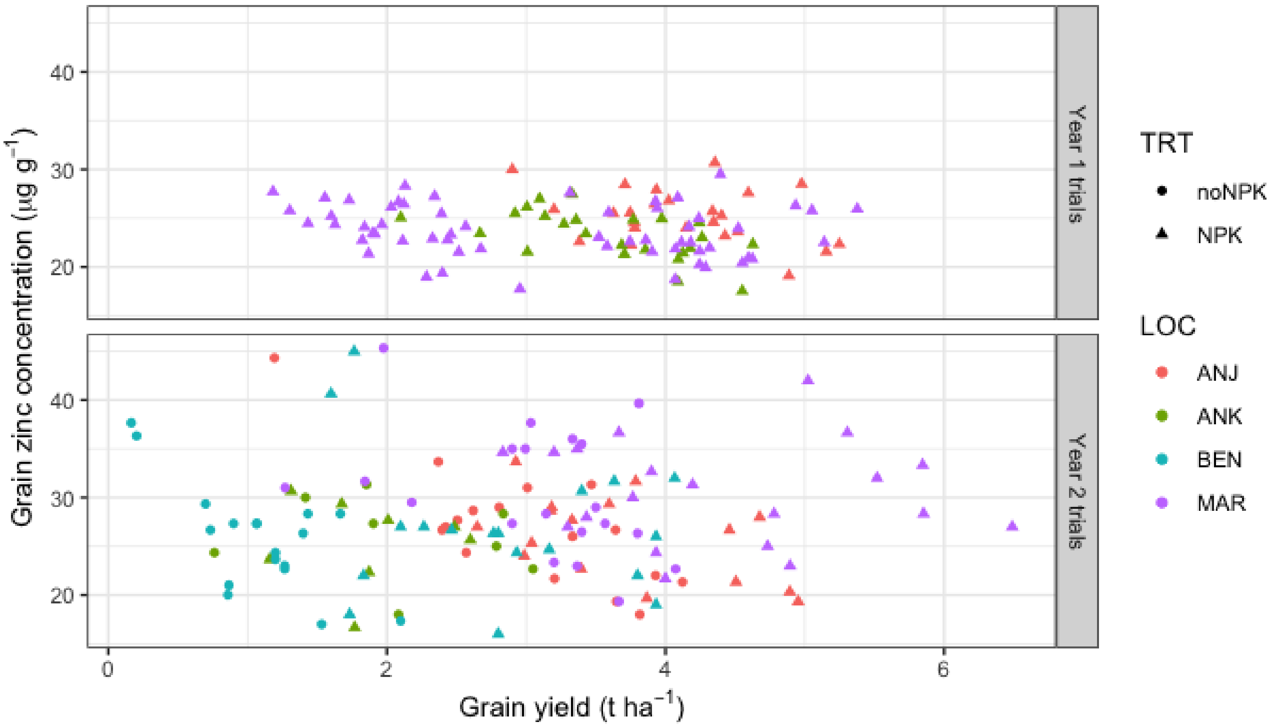

Supplementary Figure 6. Relationship between grain yield and grain zinc concentration in the two year trials. The TRT are the two fertilizer treatments NS the LOC are the locations where the trials were conducted.

Supplementary Table 1. Average performance of the germplasm groups (means ± sd) for grain yield (GY) and grain zinc concentration (grain [Zn]) measured in the four environments of year 1 trials.

Site	Group	GY	grain [Zn]
ANJ1	X265	4.91 ± 0.80	19.10 ± 0.71
	BF-Lines	4.05 ± 0.86	25.70 ± 2.85
	IR64	5.10 ± 0.77	21.56 ± 0.93
	IR68144	4.41 ± 1.03	25.70 ± 5.09
	all	4.43 ± 0.94	23.61 ± 3.59
ANK1	X265	4.59 ± 0.48	17.51 ± 1.78
	BF-Lines	3.58 ± 0.67	23.45 ± 2.31
	IR64	3.72 ± 0.60	21.36 ± 1.20
	IR68144	2.66 ± 0.28	26.75 ± 2.05
	all	3.78 ± 0.75	21.96 ± 3.15
MAR1a	X265	3.16 ± 0.88	17.50 ± 3.54
	Local	2.37 ± 0.71	21.20 ± 2.35
	BF-Lines	1.99 ± 0.45	25.11 ± 2.92
	IR64	2.27 ± 0.67	22.92 ± 2.57
	IR68144	1.35 ± 0.37	25.00 ± 2.83
	all	2.11 ± 0.59	23.96 ± 3.28
MAR1b	X265	4.50 ± 1.08	20.50 ± 3.54
	Local	4.13 ± 0.66	20.50 ± 1.72
	BF-Lines	4.31 ± 0.82	24.20 ± 2.82
	IR64	4.12 ± 0.76	22.50 ± 1.17
	IR68144	3.47 ± 0.36	27.50 ± 2.12
	all	4.23 ± 0.79	23.37 ± 2.89

Supplementary Table 2. Performance (mean and sd of BLUEs across environment), AMMI stability value (ASV) and ranked ASV (rASV) for grain yield (GY) and grain [Zn] across all environments considered in year 1 (Y1) and year 2 (Y2) trials, taken individually or combined (only the conditions with NPK fertilization).

		GY				grain [Zn]			
	Genotype	mean	sd	ASV	rASV	mean	sd	ASV	rASV
Y1 NPK	BF001	3.69	0.85	0.36	10	23.13	1.20	0.76	8
	BF008	3.72	1.13	0.11	3	24.41	2.08	0.61	7
	BF011	3.46	1.29	0.67	16	25.16	1.35	1.40	19
	BF012	3.89	1.38	0.80	19	23.17	1.22	1.39	18
	BF014	3.37	0.89	0.27	6	22.71	0.50	0.57	4
	BF015	3.30	1.10	0.61	15	22.74	0.85	0.40	2
	BF021	3.45	0.84	0.47	13	26.22	2.25	2.12	22
	BF035	3.29	0.68	0.77	18	24.23	2.16	1.74	21
	BF045	2.88	0.94	0.34	9	24.72	1.03	0.59	5
	BF050	3.70	1.21	0.46	12	22.24	1.68	0.76	9
	BF051	3.56	1.02	0.50	14	23.46	2.17	1.20	15
	BF054	3.61	1.39	1.30	24	21.58	2.07	0.61	6
	BF055	3.64	1.61	0.85	23	25.41	0.77	1.22	16
	BF060	3.48	0.91	0.10	2	26.68	1.44	1.06	11
	BF091	3.89	0.93	0.25	4	23.13	2.99	2.33	23
	BF105	2.83	1.08	0.72	17	25.45	3.40	3.09	24
	BF109	3.18	0.99	0.09	1	27.37	0.79	0.38	1
	BF110	3.78	1.18	0.31	8	27.13	2.58	1.60	20
	BF111	3.23	0.91	0.37	11	26.55	0.90	0.44	3
	BF153	2.50	1.07	0.26	5	25.43	1.94	1.14	14
	BF156	4.21	1.61	0.84	21	25.21	2.65	1.29	17
	IR64	3.83	1.17	0.85	22	22.07	0.76	1.01	10
	IR68144	3.09	1.13	0.80	20	26.37	1.11	1.12	13
	X265	4.24	0.87	0.30	7	18.70	1.34	1.08	12
Y2 noNPK	BF055	2.00	0.83	0.68	1	27.74	1.07	2.51	5
	BF109	2.36	1.01	1.48	6	31.62	4.37	3.43	7
	BF110	2.42	1.08	0.83	2	28.29	3.56	2.59	6
	BF156	2.30	1.05	2.68	7	26.31	2.60	1.04	1
	IR64	2.63	1.29	0.91	3	22.76	2.11	1.36	2
	IRIS_313-10114	2.44	0.96	1.18	4	31.60	3.65	1.90	4
	X265	2.90	0.98	1.35	5	19.33	2.15	1.72	3
Y2 NPK	BF055	2.91	0.81	1.02	6	28.10	1.60	2.32	7
	BF109	3.29	1.12	0.50	4	31.00	3.55	1.69	5
	BF110	3.58	1.35	1.17	7	27.38	4.23	2.19	6
	BF156	3.57	0.90	0.06	2	27.05	1.78	0.94	2
	IR64	3.14	1.06	0.04	1	23.14	3.07	1.17	3

	IRIS_313-10114	3.22	1.27	0.95	5	31.38	2.68	1.63	4
	X265	3.74	1.12	0.48	3	19.48	2.52	0.41	1
Y1/Y2 NPK	BF055	3.18	1.14	1.11	5	27.12	1.88	3.36	5
	BF109	3.25	1.03	0.71	2	29.68	3.34	2.04	4
	BF110	3.65	1.23	1.15	6	27.29	3.57	3.41	6
	BF156	3.80	1.17	0.74	4	26.38	2.20	0.88	2
	IR64	3.39	1.10	0.56	1	22.75	2.47	0.89	3
	X265	3.92	1.02	0.72	3	19.19	2.12	0.78	1

Supplementary Table 3. Average performance of all genotypes tested for grain yield (GY) and grain zinc concentration (grain [Zn]) (means ± sd) in the seven environments and two fertilizer conditions (noNPK and NPK) in year 2 trials.

Site	GY		grain [Zn]	
	noNPK	NPK	noNPK	NPK
ANJ2a	2.57 ± 0.75	3.31 ± 0.69	28.00 ± 7.79	27.89 ± 7.28
ANJ2b	3.50 ± 0.62	4.12 ± 0.79	27.44 ± 6.28	28.74 ± 9.36
ANK2	2.09 ± 0.80	1.86 ± 0.57	27.67 ± 6.70	27.41 ± 7.34
BEN2a	1.22 ± 0.55	3.31 ± 1.19	26.30 ± 5.73	28.19 ± 6.63
BEN2b	1.07 ± 0.42	2.36 ± 0.77	25.26 ± 5.80	25.74 ± 8.18
MAR2a	3.29 ± 0.52	3.54 ± 0.60	28.71 ± 6.07	30.47 ± 5.26
MAR2b	2.90 ± 1.32	5.26 ± 0.80	32.52 ± 7.43	30.70 ± 5.74

Supplementary Table 4. Individual performance of the 9-10 genotypes (lsmeans, n=3) for grain yield (GY) and grain zinc concentration (grain [Zn]) measured in the seven environments and two fertilizer conditions (noNPK and NPK) in year 2 trials. Means followed by different letters are significantly different (Tukey test at alpha = 0.05) within an environment and a treatment.

		GY				grain [Zn]			
Site	Genotype	noNPK		NPK		noNPK		NPK	
ANJ2a	X265	3.20	bc	3.87	ab	21.7	ab	19.7	a
	Local	3.82	c	4.51	b	18.0	a	21.3	ab
	BF055	2.42	b	3.33	ab	27.0	abc	27.7	bc
	BF109	2.62	b	3.18	a	28.7	bc	28.7	bc
	BF110	2.40	b	3.04	a	26.7	abc	25.3	ab
	BF156	2.51	b	2.65	a	27.7	bc	27.0	abc
	IRIS_313-9368	1.19	a	na		44.3	d	43.7	d
	IRIS_313-10114	2.37	b	2.93	a	33.7	c	33.7	c
	IR64	2.57	b	2.99	a	24.3	abc	24.0	ab
ANJ2b	X265	3.65	ab	4.89	c	19.3	a	20.3	a
	Local	4.12	b	4.95	c	21.3	a	19.3	a
	BF055	2.81	a	3.18	a	29.0	bc	29.0	c
	BF109	3.01	ab	3.60	abc	31.0	c	29.3	c
	BF110	3.33	ab	4.68	bc	26.0	b	28.0	bc
	BF156	3.64	ab	4.46	abc	26.7	b	26.7	bc
	IRIS_313-9368	na		na		40.3	d	51.7	d
	IRIS_313-10114	3.47	ab	3.79	abc	31.3	c	31.7	c
	IR64	3.93	ab	3.40	ab	22.0	a	22.7	ab
ANK2	X265	2.08	bcd	1.77	ab	18.0	a	16.7	a
	Local	0.76	a	1.16	a	24.3	bc	23.7	bc
	BF055	1.90	bc	2.01	ab	27.3	bcd	27.7	bcd
	BF109	1.85	bc	1.68	ab	31.3	d	29.3	cd
	BF110	2.84	cd	2.60	b	28.3	bcd	25.7	bcd
	BF156	2.79	cd	2.49	b	25.0	bc	27.0	bcd
	IRIS_313-9368	na		na		42.0	e	43.7	e
	IRIS_313-10114	1.47	ab	1.31	a	30.0	cd	30.7	d
	IR64	3.05	d	1.87	ab	22.7	ab	22.3	ab
BEN2a	X265	2.10	d	3.93	c	17.3	a	19.0	a
	Local	1.07	abc	4.07	c	27.3	c	32.0	d
	BF055	0.73	ab	2.27	ab	26.7	bc	27.0	bcd
	BF109	1.43	bcd	3.63	bc	28.3	c	31.7	d
	BF110	0.70	ab	3.17	bc	29.3	c	24.7	abc
	BF156	1.27	abc	3.93	c	23.0	abc	26.0	bcd
	IRIS_313-9368	0.23	a	1.60	a	36.3	d	40.7	e
	IRIS_313-10114	1.67	cd	3.40	bc	28.3	c	30.7	cd
	IR64	0.87	abc	3.80	bc	20.0	ab	22.0	ab

BEN2b	X265	1.53	c	2.80	a	17.0	a	16.0	a
	Local	1.27	bc	2.80	a	22.7	abc	26.3	c
	BF055	1.07	bc	2.10	a	27.3	c	27.0	c
	BF109	0.90	b	2.47	a	27.3	c	26.7	c
	BF110	1.20	bc	1.83	a	23.7	bc	22.0	abc
	BF156	1.20	bc	2.93	a	24.3	bc	24.3	bc
	IRIS_313-9368	0.17	a	1.77	a	37.7	d	45.0	d
	IRIS_313-10114	1.40	c	2.77	a	26.3	bc	26.3	c
	IR64	0.87	b	1.73	a	21.0	ab	18.0	ab
MAR2a	X265	3.67	a	4.00	a	19.3	a	21.7	a
	Local	3.20	a	3.93	a	23.3	ab	24.3	ab
	BF021	3.57	a	3.37	a	27.3	bc	35.0	de
	BF055	2.90	a	3.30	a	27.3	bc	27.0	abc
	BF109	2.90	a	3.20	a	35.0	d	34.7	de
	BF110	3.50	a	3.90	a	29.0	c	32.7	cde
	BF156	3.40	a	3.77	a	26.5	bc	30.0	bcd
	IRIS_313-9368	3.03	a	3.67	a	37.7	d	36.7	e
	IRIS_313-10114	3.33	a	2.83	a	36.0	d	34.7	de
	IR64	3.37	a	3.43	a	23.0	ab	28.0	bc
MAR2b	X265	4.07	a	4.89	a	22.7	a	23.0	a
	Local	3.14	a	6.08	a	28.3	ab	27.0	ab
	BF021	1.84	a	5.85	a	31.7	abc	28.3	abc
	BF055	2.18	a	4.20	a	29.5	abc	31.3	bcd
	BF109	3.81	a	5.31	a	39.7	cd	36.7	de
	BF110	3.00	a	5.85	a	35.0	bcd	33.3	cd
	BF156	1.27	a	4.78	a	31.0	abcd	28.3	abc
	IRIS_313-9368	1.98	a	5.02	a	45.3	d	42.0	e
	IRIS_313-10114	3.40	a	5.52	a	35.5	bcd	32.0	bcd
	IR64	3.80	a	4.73	a	26.3	ab	25.0	a
